# Case Report: Optic neuritis as the initial presentation of Orientia tsutsugamushi infection detected by metagenomic next-generation sequencing

**DOI:** 10.3389/fimmu.2023.1129246

**Published:** 2023-05-15

**Authors:** Chuan-bin Sun, Zhiqiong Ma, Zhe Liu

**Affiliations:** ^1^Eye Center, Second Affiliated Hospital of Zhejiang University School of Medicine, Hangzhou, China; ^2^Department of Ophthalmology, Zhejiang Provincial People’s Hospital, People’s Hospitalof Hangzhou Medical College, Hangzhou, China

**Keywords:** scrub typhus, optic neuritis, metagenomic next-generation sequencing, Orientia tsutsugamushi, anticardiolipin antibody, beta-2-glycoprotein-I antibody

## Abstract

Scrub typhus is an acute febrile illness caused by Orientia tsutsugamushi infection, and typically manifested as fever, eschar, lymphadenopathy, rash, and other flu-like signs. Ocular involvement was not uncommon, and mostly occurred at post-fever or recovery stage in scrub typhus cases. We hereby report a case of scrub typhus presenting as unilateral optic neuritis (ON). A 56-year-old man going wild fishing nearly every day complained of a blurred vision and an inferior visual field defect in the right eye two or three days after an insect-bite like shin induration in his left leg. He was diagnosed as ON, and treated with dexamethasone in the local hospital. Unfortunately, his right eye vision progressively deteriorated during steroid therapy. Three days after steroid therapy ceased, he suffered from a high fever and painful subcutaneous masses in the left groin. Peripheral blood test by metagenomic next-generation sequencing (mNGS) was positive for Orientia tsutsugamushi, but negative for other pathogens. The diagnosis was then revised to scrub typhus and ON. His systemic symptoms rapidly disappeared after oral doxycycline and omadacycline therapy. However, his right eye vision continuously deteriorated to hand motion. Further serum tests for aquaporin 4-IgG antibody and myelin oligodendrocyte glycoprotein-IgG antibody were both negative, but for anticardiolipin IgM and beta-2-glycoprotein-I IgM were both positive. The patient’s right eye vision gradually improved after doxycycline combined with steroid pulse therapy. Our case indicates that ON in scrub typhus cases may present as a parainfectious inflammation, and that mNGS is a useful and valuable method for early diagnosis of scrub typhus.

## Introduction

Scrub typhus is an acute febrile illness caused by Orientia tsutsugamushi infection which was transmitted to humans through the bite of the larval form of a trombiculid mite (1, 2).

Scrub typhus mainly occurs in rural and jungle areas in Southeast Asia, India, Australia, Japan, and China, and is typically manifested as fever, eschar, lymphadenopathy, rash, and other flu-like signs (1–5). Previous investigations revealed that ocular involvement including conjunctivitis, uveitis, retinitis, retinal vasculitis, and optic neuritis (ON) was not uncommon, and mostly occurred at post-fever or recovery stage in scrub typhus cases (6–12). As we know, ON as the initial presentation of scrub typhus has not been reported in literature. We herein report a case of scrub typhus presenting as unilateral ON, and followed by typical scrub typhus manifestations such as high fever, eschar, lymphadenopathy, and skin rash. Orientia tsutsugamushi infection was confirmed in a peripheral blood sample by metagenomic next-generation sequencing (mNGS), whereas serum testes for other pathogens, as well as aquaporin 4-IgG antibody and myelin oligodendrocyte glycoprotein-IgG antibody were all negative.

## Methods

Detailed medical records including systemic and ophthalmic medical history, complete ophthalmic examinations, color fundus photography, B-type ultrasound, visual field test, serum laboratory tests, as well as orbital MRI at presentation and follow-ups, were collected and analyzed. Cell-based indirect immunofluorescence assay (CBA) using HEK-293 cells as substrate was performed to detect serum aquaporin 4-IgG antibody (AQP-4 Ab), myelin oligodendrocyte glycoprotein-IgG antibody (MOG-Ab), and glial fibrillary acidic protein-IgG antibody (GFAP-Ab). Tissue-based indirect immunofluorescence assay (TBA) using monkey cerebellum tissue sections as substrate was also performed to detect possible serum antineuronal and antiglial cell autoantibodies following procedures reported in previous literature (13, 14). This study was conducted according to the tenets of the Declaration of Helsinki. Informed consent was obtained from the patient. Institutional review board approvals were obtained from Second Affiliated Hospital of Zhejiang University School of Medicine.

### mNGS test

A peripheral blood sample was collected and tested by mNGS as described in previous reports (2–5). The Blood sample was stored at 4°C, and transmitted on dry ice. DNA was extracted and purified using the MicroElute Genomic DNA Kit (DP316; TIANGEN, Beijing, China). DNA concentration and quality were checked through Qubit and agarose gel electrophoresis. Then, DNA libraries was constructed using QIAseq™ Ultralow Input Library Kit. The concentration and quality of libraries were checked using Qubit and agarose gel electrophoresis. Qualified libraries with different barcode labeling were pooled together and sequenced on an Illumina Nextseq platform. After obtaining the sequencing data, high-quality data were generated after filtering out the adapter, low quality, low complexity, and shorter reads (<35bp). Human reads were removed by mapping reads to the human reference genome using SNAP software. The remaining reads were finally blasted against Microbial Genome Databases (ftp://ftp.ncbi.nlm.nih.gov/genomes/) using Burrows-Wheeler Alignment. The database collected microbial genomes from NCBI and contains more than 20,000 microorganisms, including 11910 bacteria, 7103 viruses, 1046 fungi and 305 parasites. Organisms with sequences in low abundance and organisms not known to cause uveitis were subtracted. Finally, the pathogenic microbial compositions of the sample were determined, and the probable pathogens and their mapped reads numbers were reported.

## Case description

A 56-year-old man going wild fishing nearly every day complained of a blurred vision and an inferior visual field defect in the right eye for one day. At presentation, he denied any systemic symptoms, but mentioned an insect-bite-like skin induration in his left leg two or three days prior to his visual symptom onset. His past medical history was remarkable. Ophthalmic examination in the medical record of local hospital showed a visual acuity of 20/20, no conjunctival injection, clear cornea, quiet anterior chamber, normal pupil size and light reflex, and swollen optic disc in the right eye, and normal left eye. He was diagnosed as ON in the right eye, immediately admitted to the local hospital, and treated with intravenous injection of dexamethasone 10 mg once per day for seven days.

However, his right eye vision progressively deteriorated to 20/50 during steroid therapy. Hence, the patient refused further steroid therapy. Unfortunately, 3 days after the cease of steroid therapy, he suffered from a high fever of 39°C and painful subcutaneous masses in the left groin. He then came to other hospitals for consultation, where physical examination revealed an eschar on the lateral skin of his left leg, and enlarged lymph nodes in the left groin which was confirmed by B-type ultrasound. His white blood cell count was 10.5 x 10 (9) cells/L with neutrophils 88.3% (higher), lymphocytes 6.8% (lower), monocytes 4.6%, basophil 0.3%, and eosinophils 0%, red blood cell count 4.76 x 10 (12) cells/L, platelet 118 x 10 (9) cells/L. Activated partial thromboplastin time was 28 seconds (normal), thrombin time 18 seconds (normal), prothrombin time 10.4 seconds (normal), fibrinogen 2.13 g/L (normal), and D-Dimer 0.52 mg/L (normal). Erythrocyte sedimentation rate was 3 mm/hour (normal), C reactive protein was 12 mg/L (higher). Serum IgM was 3.333 g/L(higher), whereas serum IgG, IgA, Complement C3 and C4 were all within normal limits. Serum tests for pathogens including treponema pallidum, mycobacterium tuberculosis, herpes simplex virus, varicella-zoster virus, cytomegalovirus, hepatitis B virus, hepatitis C virus, HIV, Dengue virus, novel bunyavirus, and toxoplasma gondii, as well as antinuclear antibodies, and antineutrophil cytoplasmic antibodies, were all negative. CBA revealed a triple negative test result for serum AQP-4 Ab, MOG-Ab, and GFAP-Ab. Serum test for Orientia tsutsugamushi by mNGS was positive. He was then diagnosed as scrub typhus and ON, and treated with doxycycline 100 mg twice per day, and omadacycline 300 mg per day for 6 days. His fever and diffuse shin rash which appeared on the day of antibiotic therapy rapidly resolved after treatment. However, his vision in the right eye continuously deteriorated to hand motion.

**Figure 1:**
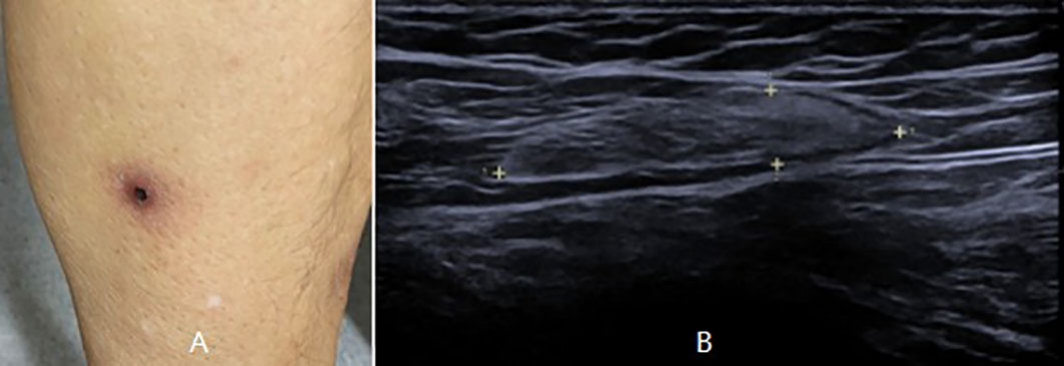
Physical examination showed an eschar on the left leg **(A)**, and B-type ultrasound showed enlarged lymph nodes in the left groin **(B)**.

**Figure 2:**
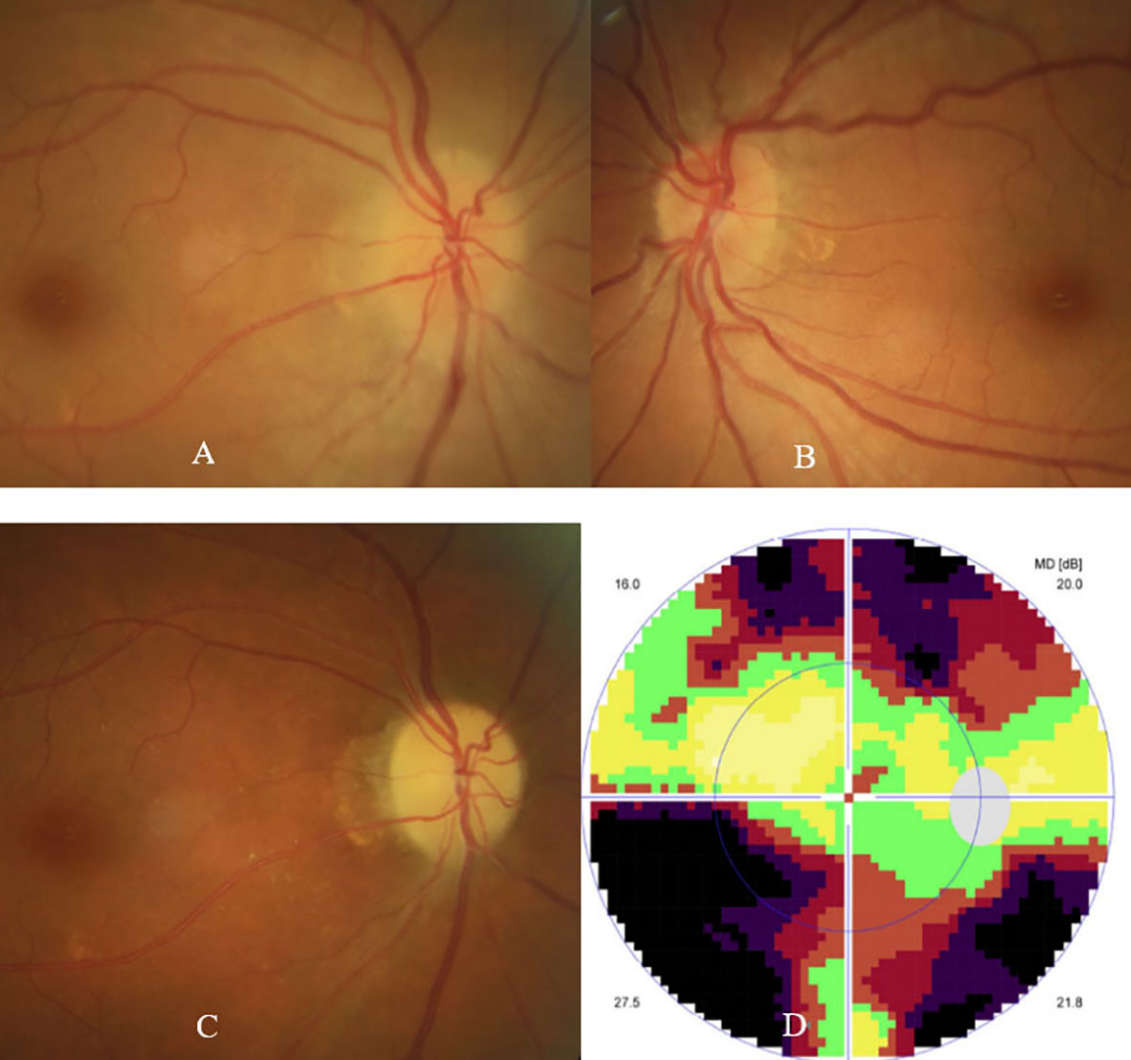
Fundus photographs showed swollen optic disc in the right eye **(A)**, and normal fundus in the left eye **(B)** at presentation. Fundus photograph showed pale optic disc **(C)**, and visual field test showed tunnel field **(D)** in the right eye at 11 weeks follow-up.

**Figure 3:**
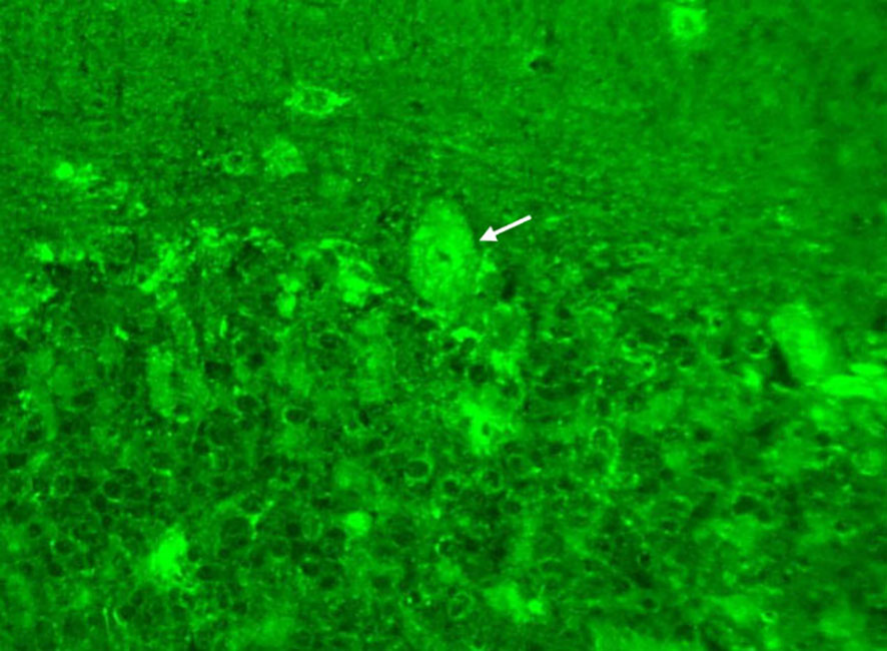
Tissue-based indirect immunofluorescence assay showed granular intracytoplasmic and circular perinuclear immunofluorescence staining in Purkinje cells (arrow) of monkey cerebellum tissues incubated with the serum samples (diluted as 1:100) of the patient.

**Figure 4:**
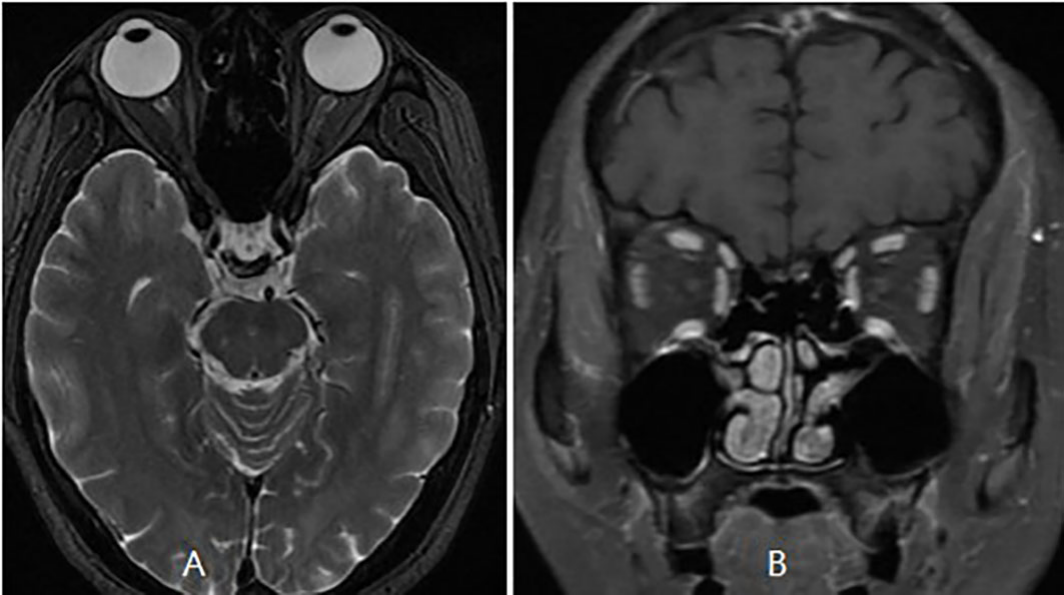
Orbital MRI showed normal optic nerves **(A)** with no enhancement **(B)**.

The patient was then referred to our neuro-ophthalmology clinic for further consultation. At presentation, physical examination confirmed the skin eschar on the left leg (**Figure 1A**), and B-type ultrasound revealed enlarged lymph nodes in both groins (**Figure 1B**). Ophthalmic examination revealed his right eye had a visual acuity of light perception, clear cornea, quiet anterior chamber, dilated pupils of 6 mm with sluggish light reflex, clear lens, and swollen optic disc (**Figure 2A**), and his left eye was normal (**Figure 2B**). Intraocular pressure was normal in both eyes. Further serum tests showed anticardiolipin IgM antibody of 154.4 GPL/ml (higher), beta-2-glycoprotein-I IgM antibody of 112.3 SMU/ml (higher), and C reactive protein 1.1 mg/L (normal). TBA revealed granular intracytoplasmic and circular perinuclear immunofluorescence staining in Purkinje cells (**Figure 3**, arrow) of monkey cerebellum tissues when they were incubated with the serum samples (diluted as 1:100) of the patient. Orbital MRI showed normal optic nerves with no enhancement (**Figures 4A, B**).

His diagnosis of unilateral ON secondary to scrub typhus was confirmed, and sequential intravenous methylprednisolone 500 mg per day for 3 days, 240 mg per day for 3 days, and 120 mg per day for 3 days, tapered by oral prednisone, together with doxycycline 100 mg twice per day, were prescribed. His serum titers of anticardiolipin IgM and beta-2-glycoprotein-I IgM antibodies decreased to 88.6 GPL/ml and 66.0 SMU/ml respectively one week later. Another one month later (11 weeks after the onset of ON), his right eye vision was gradually improved to 20/80, ophthalmic examination showed a pale optic disc (**Figure 2C**), and visual field test showed a tunnel field (**Figure 2D**) in the right eye. Repeated serum test revealed a further decrease in titers of anticardiolipin IgM and beta-2-glycoprotein-I IgM antibodies to 25.5 GPL/ml and 26.5 SMU/ml, respectively. The patient was followed up by telephone five months after the onset of ON, and he reported a stable visual acuity in the right eye, though steroid and doxycycline therapy already ceased.

## Discussion

Scrub typhus is an acute febrile illness characterized by ulceration or eschar at the bite site, fever, skin rash, and lymphadenopathy. Patients with scrub typhus is easily diagnosed based on a typical skin eschar and a recent outdoor experience in rural or jungle areas. However, as for atypical scrub typhus cases without skin eschar at presentation, early diagnosis of scrub typhus is quite challenging (3, 4). Hence, direct laboratory evidence of Orientia tsutsugamushi DNA or antigen in patients’ body liquid samples is of great importance for the early diagnosis of scrub typhus, especially for the atypical ones. Although Weil Felix test is a simple and unexpensive method to detect rickettsia infection (12), this test is currently not available in many hospitals including ours. Recently, mNGS is proved to be a sensitive and valuable method for simultaneous detection of thousands of pathogens including rickettsia (2–5). Our finding in this study gives further support for the clinical usage of mNGS in rickettsia detection.

Ocular involvement in scrub typhus cases mostly occurred at its post-fever or recovery stage, and was predominantly manifested as mild conjunctivitis, uveitis, retinitis or retinal vasculitis (6–17). However, ON was not uncommon in scrub typhus cases (6, 9, 18–20). One interesting finding in our case is that ON occurred prior to fever and other-flu-like symptoms, which, as the best of our knowledge, has not been reported in literature. The explanation may exist in that the patient was immediately treated with dexamethasone one day after his visual symptom onset, since steroid such as dexamethasone can significantly suppress fever and other-flu-like symptoms. This presumption was supported by the following finding that high fever occurred three days after dexamethasone therapy ceased in this case.

The pathogenesis of ON after Orientia tsutsugamushi infection is still not clear. Direct bacterial invasion, post-infectious immune−mediated inflammation, or even induction of causative ON biomarkers such as AQP-4Ab are proposed as the mechanism of ON after Orientia tsutsugamushi infection (18–24). Some previous investigations proposed that direct bacterial invasion might play a major role in the pathogenesis of ON based on their clinical experience of rickettsial retinitis therapy (18, 19). However, considering that ON mostly occurred at the post-fever or recovery stage of the natural course of scrub typhus, and showed good response to steroid combined with antibiotic agents therapy, many investigators preferred an immune-mediated to a direct invasion mechanism (19, 20).

As for this case in our study, visual field defect in ON eye occurred early (2 or 3 days after possible insect bite), visual acuity progressively deteriorated after sequential steroid therapy alone and doxycycline therapy alone, but gradually improved after doxycycline combined with steroid pulse therapy, and finally permanent vision and visual filed defects were left at five-month follow-up, indicating that both early direct invasion and subsequent parainfectious immune-mediated mechanism are involved in the pathogenesis of ON in this case (19–23).

Although a causative ON biomarker such as AQP-4Ab was occasionally reported in ON case with scrub typhus (24), no currently identified causative ON biomarkers such as AQP-4Ab and MOG-Ab were detected in our case. Previous investigations revealed that pathogen infection could induce the production of anticardiolipin antibody and (or) beta-2-glycoprotein-I antibody (25, 26). In this study, serum titers of serum anticardiolipin and beta-2-glycoprotein-I IgM antibodies were detected high at the early stage, and then progressively decreased during the natural course of ON after Orientia tsutsugamushi infection, indicating that the production of anticardiolipin and beta-2-glycoprotein-I antibodies was probably induced by Orientia tsutsugamushi infection.

It was reported that anticardiolipin and beta-2-glycoprotein-I antibodies may induce an obstructive vasculitis or an immune-mediated ON (25–28). Considering that no retinal hemorrhage or edema occurred in this case, serum anticardiolipin and beta-2-glycoprotein-I antibodies may play a role in the pathogenesis of ON, or they are just epiphenomenon antibodies after Orientia tsutsugamushi infection in our case. Moreover, TBA revealed an intracellular antineuronal autoantibody in the serum of the patient, but no responsible autoantibodies such as antinuclear antibodies were detected in this study. Hence, more investigations including paraneoplastic syndrome and autoimmune encephalitis autoantibody test need to be done to exclude whether there exists a currently unknown antineuronal autoantibody in this case (14, 27, 29).

## Data availability statement

The original contributions presented in the study are included in the article/Supplementary Material. Further inquiries can be directed to the corresponding author.

## Ethics statement

The studies involving human participants were reviewed and approved by Second Affiliated Hospital of Zhejiang University School of Medicine. The patients/participants provided their written informed consent to participate in this study. Written informed consent was obtained from the individual(s) for the publication of any potentially identifiable images or data included in this article.

## Author contributions

C-bS and ZL design and wrote the article, C-bS and ZM collected and analyzed the clinical and laboratory data. All authors contributed to the article and approved the submitted version.
